# Brainstem Auditory Evoked Potentials in Boys with Autism: Still Searching for the Hidden Truth

**Published:** 2015

**Authors:** Athina VERVERI, Euthymia VARGIAMI, Vassiliki PAPADOPOULOU, Dimitrios TRYFONAS, Dimitrios ZAFEIRIOU

**Affiliations:** 1 Department of Pediatrics, Medical School, Aristotle University of Thessaloniki, Thessaloniki, Greece; 2 Department of Child Psychiatry, Hippokration Hospital, Thessaloniki, Greece

**Keywords:** Brainstem Auditory Evoked Potentials, Autism, Auditory, Brainstem

## Abstract

**Objective:**

Brainstem auditory evoked potentials (BAEPs) have long been utilized in the investigation of auditory modulation and, more specifically, auditory brainstem functions in individuals with autism. Although most investigators have reported significant abnormalities, no single BAEPs pattern has yet been identified. The present study further delineates the BAEPs deficits among subjects with autism.

**Materials & Methods:**

BAEPs were recorded in 43 male patients, aged 35–104 months, who underwent standard evaluations after receiving a diagnosis of autism. The control group consisted of 43 age-matched typically developing boys. The study took place in a tertiary neurodevelopmental center over a period of two years.

**Results:**

The mean values of all absolute and/or interpeak latencies were longer in patients when compared to controls, albeit the differences were not significant for any of the parameters. Prolonged or shortened absolute/interpeak latencies (control group mean ± 2.5SD) were unilaterally or bilaterally identified in 33% of patients, compared to 9% of controls. The most frequent findings included prolongation of absolute latencies I, V and III, followed by shortening of interpeak latency I-V. In addition, abnormalities (either shortening or prolongation) of absolute latencies I and V, as well as interpeak latency I-V, were significantly more common among patients. Taken together, BAEPs in 23% of patients were indicative of a clinically abnormal response in 32% of patients.

**Conclusion:**

As can be easily concluded, BAEPs abnormalities characterize only a subset of subjects with autism, who may be important to identify clinically. The latter individuals may benefit from targeted intervention to utilize brainstem plasticity.

## Introduction

Autism spectrum disorders (ASD) constitute a single continuum of neurodevelopmental disabilities with multifactorial etiology and significant individual variability according to the recently published Diagnostic and Statistical Manual of Mental Disorders V (DSM-V) (1). ASDs are characterized by a rising prevalence, currently amounting to 11/1000 (2), but expected to slightly decrease due to DSM-V changes in the diagnostic criteria. The latter changes mainly include rearrangement of criteria into two, instead of three areas (social communication/ interaction and restricted, repetitive behaviors), merging of all previous sub-diagnoses under the same spectrum, and inclusion of sensory abnormalities among the symptoms related to the second criterion (restricted, repetitive behaviors) (3). Sensory dysfunction is defined as “being sensory sensitive or having a low threshold for response to stimuli” (4). It involves several sensory modalities that produce auditory, visual, touch, and oral symptoms in more than three-quarters of preschool children with autism (5). A disruption of sensory modulation with both under and over-reactivity to sound has given rise to a series of auditory perception and processing studies. Brainstem auditory evoked potentials (BAEPs) have been widely utilized in this field. Their qualities include simultaneous assessments of both hearing level and brainstem function along with no requirement for co-operation or conscience. Initial BAEPs work in the 1970s had produced rather promising results by identifying a wide variety of discrepancies in the autism population. However, these early studies were characterized by a substantial lack of consistency, leading to the assumption that BAEPs alteration reflects samples characteristics, rather than ASDs (6). Once initial methodological issues had been resolved, reports of abnormal BAEPs reemerged in the early 1990s. Reported abnormalities included isolated or combined prolongation of different absolute and interpeak latencies (IPLs). Although results remained abnormal, they still lacked replicability across different study groups (7). Nevertheless, a number of important themes were gradually brought to light through BAEPs investigation: first, there is a solid relationship between autism and prolonged auditory transmission times that implies a role for the brainstem in autism pathogenesis. Second, auditory abnormalities occur only in a subset of participants with autism who would probably be important to identify clinically (8). Thus, interest in BAEPs investigation is justified and required on clinical and research grounds. The present study analyzes BAEPs results in a large group of Greek boys with autism and age-matched controls. Effort was undertaken to minimiaze confounding factors identified in previous studies.

## Materials & Methods

The study sample included 43 boys with autism (mean age 48 months, range 35–104 months, SD 12 months) who underwent BAEPs during their standard evaluation after receiving ASD diagnosis. The study was conducted in a tertiary neurodevelopmental center during a period of two years. Exclusion criteria comprised perinatal history of prematurity/asphyxia/ischemia and/or presence of comorbidities (genetic/metabolic syndromes or neurological diagnoses, e.g. epilepsy, cerebral palsy). Additionally, individuals who required sedation but did not respond to oral chloral hydrate were excluded from the study due to ethical considerations preventing general anesthesia. In all cases, diagnosis was posed or confirmed by a child psychiatrist in accordance to the criteria of the Diagnostic and Statistical Manual of Mental Disorders IV, Text Revision (9) whereas the severity of symptoms was assessed by the Childhood Autism Rating Scale (CARS) (10). In addition to BAEPs, the standard evaluation also included an electroencephalogram (EEG) and thyroid/genetic/metabolic testing; in selected cases, neuroimaging with brain magnetic resonance imaging (MRI) was also undertaken. The control group included 43 age-matched boys (nonsignificant mean age difference). All participants were hospitalized in the adjunctive general pediatric clinic due to urinary, gastrointestinal, and hematological disorders, among others. They had no apparent medical reason for hearing impairment and no signs of central nervous system (CNS) pathology. BAEPs were recorded on a Nihon Kohden Neuropack 4 apparatus in a soundproof and electrically shielded room at a room temperature between 24–25°C and all participants were asleep. Oral chloral hydrate was administered when required namely in the majority of patients and a small subset of controls. Electrodes were placed at Fz, ipsilateral earlobe, and contralateral earlobe (ground). Click stimuli were presented monaurally at a rate of 10 Hz and at 70 dB above normal hearing level (band pass 200–2000 Hz, speed duration 10 ms). Two recordings each consisting of 1024 sweeps were averaged. The recorded variables included absolute latencies of waves I, III, and V as well as interpeak latencies (IPLs) of waves I–III, III–V, and I–V. Before the investigation, informed consent was obtained from parents of all children with autism as well as controls. The prevalence of BAEPs abnormalities was calculated using comparison group means ± 2.5 SD as an upper/ lower limit for normal values. The established normative criteria for the latency parameters were in agreement with those widely reported in literature (11) (Table I). Comparison of independent samples was performed with the non-parametric Mann-Whitney U test whereas comparison of qualitative and quantitative variables was carried out with Chi-square/Fisher’s exact test and Spearman’s correlation, respectively. Left-right differences of both absolute and interpeak latencies were investigated by means of paired t-test.

## Results

**Fig 1:**
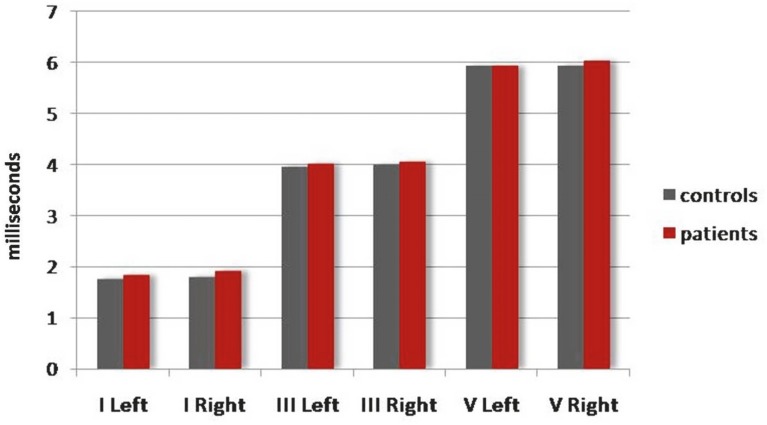
Comparison between patients with autism (red bars) and controls

Six parameters were bilaterally obtained from each participants resulting in recordings for 86 ears in each group (patient and control). Mean values of all absolute and/or interpeak latencies were longer in the patient when compared to the control, pooled recordings on both sides (Figure 1). The difference was not significant for any of the parameters (p > 0.22) (Mann-Whitney U tests). The mean (left and right) values for each individual were also prolonged in the control group (Table I), although the difference was again not significant (p > 0.30), (MannWhitney U tests). Moreover, a weak/moderate negative correlation between age and all absolute latencies was identified in the patient (rs ranging from -0.27 to -0.43, n = 43, p < 0.05) (Spearman’s correlations), but not in the control, bilateral recordings (rs < 0.19, n = 43, p>0.05) (Spearman’s correlations). On the contrary, no significant correlation was identified between BAEPs parameters and patient percentile of head circumference (rs < 0.19, n = 43, p > 0.05) (Spearman’s correlations). Prolonged or shortened absolute/interpeak latencies (control group mean ± 2.5SD) were unilaterally or bilaterally identified in 14 of 43 patients (33%) but only in 4 of 43 controls (9%). Table II details the various types of abnormalities in 86 unilateral recordings of each group. The most frequent disorders among patient recordings included the prolongation of waves I (13%) and V (9%), followed by the prolongation of wave III (8%). Among patient recordings with pathological IPLs, the most common deficit was shortening of IPL I-V (6%), which was, interestingly, two times more common than prolongation of the same wave. The prevalence of abnormalities (either shortening or prolongation) of latencies I (p = 0.005) and V (p = 0.028) as well as IPL I-V (p = 0.034) was significantly higher in the patient when compared to the control recordings (Chi-square tests). Although the rest of abnormalities also occurred more frequently among patient recordings, the prevalence differences between the two groups were not significant (p > 0.132) (Chi-square tests) (Table II). As far as an EEG is concerned, it was abnormal in 6/43 children with autism (generalized paroxysmal activity in four and diffuse slowing of basic rhythm in two), whereas MRI was undertaken in 32 patients and was abnormal in six. Four individuals manifested congenital dysplasia and the remaining two had myelin delay. Among subjects with autism, the presence of MRI findings was significantly correlated with longer absolute latency III and IPL I-III on the right side (p = 0.033 and 0.007, respectively) (Mann-Whitney U tests), whereas the identification of EEG abnormalities was correlated with longer IPL I-V on the right side (p = 0.041) (Mann-Whitney U tests). There was no significant correlation between CARS scores and the outcome of the various BAEPs parameters (rs < 0.19, n = 43, p > 0.05) (Spearman’s correlations). Taken together, BAEPs of 10/43 children with autism (23%) and 2/43 controls (4.7%) exhibited pathological values indicative of a clinically abnormal response. As far as the patient group is concerned, the deficit was apparently conductive in eight cases and sensorineural in the remaining two (Table III). The prevalence of abnormal BAEPs was significantly higher in the patient group (p = 0.026) (Chi-square test). Abnormal BAEPs in patients were not correlated with age (p = 0.93) (MannWhitney U test), CARS score (p = 0.83) (Mann-Whitney U test), EEG findings (p = 0.57) (Fisher’s exact test), or MRI abnormalities (p = 0.31) (Fisher’s exact test). Finally, there was no significant difference between left and right side absolute and/or interpeak latencies in either the patient or the control group (p > 0.05) (paired t-tests).

(grey bars) for the mean values of different latencies (in milliseconds)

**Table 1:** Normative Values in Literature and Mean (Left And Right) Values in Control and Patient Groups for Different Baeps Parameters (in Milliseconds): A Comparison of Mean Latencies between the Control and Patient Groups

		**I**	**III**	**V**	**IPL I-III**	**IPL III-V**	**IPL I-V**
**Normative values in literature** *	**Mean**	1.7	3.8	5.7			4.0
	**SD**	0.2	0.2	0.2			0.2
	**Upper Limit** **	2.2	4.3	6.2			4.5
**Control group**	**Mean**	1.8	4.0	5.9	2.2	2.0	4.2
	**SD**	0.2	0.2	0.2	0.3	0.2	0.2
	**Lower limit** **	1.3	3.5	5.4	1.5	1.5	3.7
	**Upper limit** **	2.3	4.5	6.4	3.0	2.5	4.7
**Patient group**	**Mean**	1.9	4.0	6.0	2.2	2.0	4.2
	**SD**	0.3	0.4	0.4	0.3	0.3	0.3
**(Mann-Whitney U Test)** **p-value**		1.0	0.4	0.3	1.0	0.9	0.8

* Levy SR, 1997

** Upper/Lower limit: Mean ± 2.5 SD

**Table 2:** Percentages of Abnormal Baeps Parameters (Control Mean ± 2.5 SD) in 172 Pooled Recordings of both Sides (Right and Left) of Patients and Controls and Comparison of Abnormality Prevalence between the Two Groups

		**I**	**III**	**V**	**IPL I-III**	**IPL III-V**	**IPL I-V**
**Patients**	**prolonged** **shortened** **total**	11/86 0/86 11/86 (13%)	7/86 2/86 9/86 (10%)	8/86 4/86 12/86 (14%)	0/86 4/86 4/86 (5%)	4/86 2/86 6/86 (7%)	3/86 5/86 8/86 (9%)
**Controls**	**prolonged** **shortened** **total**	1/86 0/86 1/86 (1%)	2/86 1/86 3/86 (3%)	3/86 0/86 3/86 (3%)	0/86 3/86 3/86 (3%)	4/86 0/86 4/86 (5%)	1/86 0/86 1/86 (1%)
**(Fisher’s exact test) ** **p-Value**		0.005	0.132	0.028	1.0	0.746	0.034

**Table 3:** Types of BAEPs Abnormalities Identified in Patient and Control Groups

**Type of deficit**	**Patients (n = 43)**	**Controls (n = 43)**
Peripheral	8	1
Central	2	1
Total	10 (23%)	2 (4.7%)

## Discussion

The present investigation further delineates the BAEPs pattern among boys with autism aged 35–104 months old. Although BAEPs abnormalities have been repeatedly reported in patients with ASD, no single BAEPs pattern has yet to be identified. Results by different study groups are characterized by substantial inconsistency, probably owing to age and other selection differences among the samples. Reported abnormalities include significant prolongation/ shortening of some or all absolute (12-14) and/or interpeak latencies (12,15); whereas, rarely, investigators have reported no significant alteration at all (13,16). The latter finding has been replicated in the present study; although all absolute and interpeak latencies were prolonged, no statistical significance was identified. However, this was not the case with the prevalence of abnormalities as follows: certain absolute and interpeak latencies (wave I and IPL I-V on the right side) aberrations were significantly more common in the patients when compared to the control recordings. Moreover, right IPL I-V was significantly longer among participants with EEG findings and right wave III, as well as IPL I-III, was significantly longer in patients with MRI abnormalities. The prolongation of wave I has been repeatedly reported in the literature (12,13). Rosenhall et al (2003) identified a longer wave I in 24% of 101 individuals with autism. The authors postulated a cochlear or crossed olivocochlear bundle dysfunction, which may also manifest in patients with tinnitus and prolongation of wave I (12). The second significantly common abnormality in the present sample consisted of prolonged IPL I-V in 9% of patient recordings versus 1% of control recordings. IPL I-V prolongation is a rather common finding in previous studies (17-19). Maziade et al (2000) have identified significantly longer IPLs I-V and I-III in individuals with autism and their non-autistic first-degree relatives (only IPL I-III was affected in relatives). The IPL I-III abnormality in proband families has implicated genetic as well as among a variety of other neurobiological factors in BAEPs of patients with autism (17). Apart from latency prolongation, patient recordings in the present sample exhibited a high prevalence of shortened IPL I-V. The latter finding has been scarcely reported in just a few early studies (20,21), whereas it is common among patients with Down syndrome before the age of one. In the case of trisomy 21, shortening of stimulus transmission is suggestively associated with accelerated maturation or various anatomical/functional disturbances in the CNS (22). It should be highlighted that all BAEPs parameters in the present study exhibited a negative, albeit weak, correlation with age. In typically developing children, absolute latencies gradually become shorter, reaching adult values, by the age of 2 years. Latency changes are probably due to increasing myelin density in the cochlear nerve and brainstem pathways (23). The persistence of the maturation process throughout older ages may be associated with maturational defects in brainstem myelination as initially suggested by McClelland et al (1992) (24). In favor of this postulation, Harbord et al (1990) have reported diminished myelination in MRIs of children with developmental delay and abnormally prolonged BAEPs (25). In the index sample, patients with myelin delay and other MRI abnormalities exhibited significantly longer BAEPs latencies. More specifically, two of the four boys with myelin delay manifested prolongation of right wave V in one case and all absolute latencies bilaterally in the other case. In addition to disorders at the brainstem-midbrain level, BAEPs components are also affected by the descending (corticofugal) portion of the auditory system. The latter consists of efferent neurons, projecting from the auditory cortex back to the periphery (the medial geniculate body, the inferior colliculus, and the subcollicular auditory nuclei) (26), and holding an inhibitory role on the CNS input of auditory stimuli. More specifically, the efferent system stimulation leads to reduction of auditory nerve responses and basilar membrane motility (27,28), which persists, more limited, even after indirect stimulation by contralateral sound. The efferent top-down regulation has been implicated in the speech processing disorder of children with autism (29) as well as the prolongation of BAEPs latencies in normally hearing subjects when tested with contralateral white noise (30). Overall, 23% of patients manifested abnormal BAEPs compared to 4.7% of controls. This proportion is in agreement with previous ASD reports, although lower rates have been also described (31). In a review of several studies, Klin et al (1993) reported a total prevalence of 33–46% for sensorineural as well as conductive hearing disorders (32). It is noteworthy that a previous study by our Center identified a much lower rate of 11% (33). The difference between the normative values used in the two studies (mean control ± 2.5 SD herein versus normative data from literature (11) in a previous study) is subtle and unlikely to owe for the large discrepancy. It is more plausible that rate differences reflect variation in sample characteristics, such as the number of exclusion criteria applied to the index patients, in contrary to the previous consecutive and unselected sample of children with autism. An effort to diminish methodological bias was undertaken in the present study as follows the sample consisted of boys only, since female controls have shorter IPLs (so their inclusion may bias IPLs to be shorter in controls and appear longer in ASD) (6)), whereas patient age was older than three years to avoid the wave V maturation process (although the rest of parameters matured by the age of two, wave V structure is still developing until 3 years of age in typically developing children (13)). Other potential confounding factors, such as hearing loss or lateralization issues have also been controlled for by means of excluding patients with elevated BAEPs threshold and testing both ears instead of randomly choosing one. Possible limitations include the use of sedation, which was not universal to all participants; the majority of patients, in contrary to a small subset of controls, required oral chloral hydrate. The latter substance is a safe, widely used sedative in pediatric neurophysiology (34); nevertheless, its selective use in a proportion of the present sample poses potential bias in the study design. In addition, patients who did not respond to oral sedation were excluded from the study, arising issues of selection bias (e.g. participation of subjects with higher levels of cooperation and milder forms of autism). As can be easily concluded, BAEPs abnormalities characterize only a subset of patients with autism, thus being an insufficient tool for “explanation” or diagnosis of the disorder. Moreover, they are nonspecific to ASD, given their presence in language delay (14), ADHD (35), and a number of other pathologies. On the other hand, BAEPs aberrations do not generalize to all developmental disorders (6), whereas they seem to reflect a certain subgroup among children with autism (8). Identification of this subset may have important clinical as well as research implications. The brainstem bears experience-dependent plasticity especially when speech encoding is concerned. Its response to speech is “dynamic in nature and malleable by experience”; in other words, the brainstem auditory pathway is amenable to training, as evidenced by results of scalp-recorded evoked potentials (36). The role of early targeted intervention in children with abnormal BAEPs recordings is yet to be investigated. In the same direction, it would be interesting to identify the potential changes in BAEPs recordings after the application of specialized intervention. Despite the current inconsistent findings, BAEPs still sustain research interest and justify further investigation. A meta-analysis of existing literature would require great caution (due to methodological discrepancies) but may shed light into the physiological mechanisms involved in autism as well as their potential therapeutic applications in a subset of the patients. Abnormal BAEPs recordings may reflect a more pronounced brainstem dysfunction, which is amenable to early targeted intervention.

## Conflict of interest

The authors report no financial or other conflict of interest relevant to the subject of the study. The study did not receive funding of any kind. All children participated in the study on a voluntary basis, after parental consent had been obtained.
